# Electroacupuncture at ST25 corrected gut microbial dysbiosis and SNpc lipid peroxidation in Parkinson’s disease rats

**DOI:** 10.3389/fmicb.2024.1358525

**Published:** 2024-02-21

**Authors:** Xuan-ming Hu, Li-zhe-xiong Song, Zhi-zi Zhang, Xi Ruan, Hai-chang Li, Zhi Yu, Lin Huang

**Affiliations:** ^1^Key Laboratory of Chinese Medicine Rheumatology of Zhejiang Province, School of Basic Medical Sciences, Zhejiang Chinese Medical University, Hangzhou, China; ^2^Key Laboratory of Acupuncture and Medicine Research of Ministry of Education, Nanjing University of Chinese Medicine, Nanjing, China; ^3^School of Acupuncture-Moxibustion, Tuina of Nanjing University of Chinese Medicine, Nanjing, China

**Keywords:** Parkinson’s disease, electroacupuncture, gut microbiota, microbial metabolites, lipidomics, lipid peroxidation

## Abstract

**Introduction:**

Parkinson’s disease (PD) remains one kind of a complex, progressive neurodegenerative disease. Levodopa and dopamine agonists as widely utilized PD therapeutics have not shown significant positive long-term outcomes. Emerging evidences indicate that electroacupuncture (EA) have potential effects on the therapy of nervous system disorders, particularly PD, but its specific underlying mechanism(s) remains poorly understood, leading to the great challenge of clinical application and management. Previous study has shown that acupuncture ameliorates PD motor symptoms and dopaminergic neuron damage by modulating intestinal dysbiosis, but its intermediate pathway has not been sufficiently investigated.

**Methods:**

A rat model of PD was induced using rotenone. The therapeutic effect of EA on PD was assessed using the pole and rotarod tests and immunohistostaining for tyrosine hydroxylase (TH) in the substantia nigra (SN) of brain. The role of gut microbiota was explored using 16S rRNA gene sequencing and metabonomic analysis. PICRUSt2 analysis, lipidomic analysis, LPS and inflammatory factor assays were used for subsequent exploration and validation. Correlation analysis was used to identify the key bacteria that EA regulates lipid metabolism to improve PD.

**Results:**

The present study firstly reappeared the effects of EA on protecting motor function and dopaminergic neurons and modulation of gut microbial dysbiosis in rotenone-induced PD rat model. EA improved motor dysfunction (via the pole and rotarod tests) and protected TH+ neurons in PD rats. EA increased the abundance of beneficial bacteria such as Lactobacillus, Dubosiella and Bifidobacterium and decreased the abundance of Escherichia-Shigella and Morganella belonging to Pseudomonadota, suggesting that the modulation of gut microbiota by EA improving the symptoms of PD motility via alleviating LPS-induced inflammatory response and oxidative stress, which was also validated by various aspects such as microbial gene functional analysis, fecal metabolomics analysis, LPS and inflammatory factor assays and SNpc lipidomics analysis. Moreover, correlation analyses also verified strong correlations of Escherichia-Shigella and Morganella with motor symptoms and SNpc lipid peroxidation, explicating targets and intermediate pathways through which EA improve PD exercise symptom.

**Conclusion:**

Our results indicate that the improvement of motor function in PD model by EA may be mediated in part by restoring the gut microbiota, which intermediate processes involve circulating endotoxins and inflammatory mediators, SNpc oxidative stress and lipid peroxidation. The gut-microbiome - brain axis may be a potential mechanism of EA treatment for the PD.

## Introduction

Parkinson’s disease (PD), the second most common neurodegenerative disease, characterized by the accumulation of α-synuclein (α-syn) and the degeneration of dopaminergic neurons in the substantia nigra pars compacta (SNpc), with consequent motor features, such as tremor, bradykinesia postural instability, and non-motor manifestations, including constipation, sleep disorders, dementia and other symptoms involved in various functions of the gastrointestinal, central nervous system (CNS) and autonomic nervous system (ANS) (Goedert, 2015). The pathogenesis of PD relates complex polygenic factors interacting with environmental exposures. As the gastrointestinal tract represents a primary site for exposure to pathogens and gastrointestinal motility disorders is one of the main prodromal dysfunctions, the role of which the microbiome-gut-brain axis in PD has become a subject of great interest to the field (Travagli et al., 2020). Lipids in the gut microbiome play important roles in many metabolic and inflammatory diseases, and provide important signals for cell trafficking, neuronal function in the brain, and lipid raft protein function. Microbiome lipid metabolism is important for regulating brain function and may affect chronic inflammation in the brain, leading to many diseases particularly starting in the gut, such as PD.

Although the etiology of PD is generally unknown, the formation of α-syn aggregates seems to be closely associated with an altered lipid metabolism (Sarchione et al., 2021). Abnormal accumulation of lipid droplets in neurons can induce a conformational change of α-syn, which causes α-syn accumulation in human neurons (Girard et al., 2021). Phosphatidic acid (PA), especially PA (18:1/18:1), demonstrated a substantially enhancement of generation of α-helical, multimeric and PK-resistant α-syn protein (Mizuno et al., 2017). Recent data from animal models suggest that PE deficiency disrupts the homeostasis of α-syn and induces its aggregation (Wang et al., 2016), suggesting that PE may delay the pathologic progression of PD. However, the specific role of gut microbiota and lipid metabolism for PD and the factors modulating such processes along the microbiome-gut-brain axis are still largely unknown.

So far, levodopa and dopamine agonists are widely used as treatment options for PD. Nevertheless, these drugs are not showing significantly positive long-term outcomes (Raza et al., 2019). It is crucial to widening the research scope to find alternatives that can improve symptoms for PD patients in a long run. With the wide distribution of PD cases globally, more and more complementary and alternative treatments are being used for the care and control of PD. Acupuncture has promising clinical effects when used to treat dyskinesia and other related neurological disorders (Wen et al., 2021). Due to the advantages in improving the symptoms of neurological disorders and few side effects, acupuncture is considered to be a safe and useful treatment for PD (Huang et al., 2020). Although an increasing number of studies revealed acupuncture’s therapeutic effects both in clinical and animal experiments research for PD, the mechanisms of the effectiveness of acupuncture for the treatment of PD remain largely unknown (Li et al., 2022). Previous studies showed that acupuncture has neuroprotective, anti-neuroinflammatory and antiapoptotic effects in PD mice models (Park et al., 2003, 2015, 2017; Jeon et al., 2008; Kim et al., 2011a,b). It has also been shown that acupuncture enhances motor function and protects dopaminergic neurons by regulating gut microbial dysbiosis (Jang et al., 2020). However, the specific mechanisms by which acupuncture ameliorates dyskinesia and dopaminergic neurons damage in PD through gut microbiota have not been sufficiently investigated.

EA at ST25 is a commonly used clinical experience in the treatment of constipation and its efficacy has been fully confirmed (Liu et al., 2016; Wang et al., 2019). The regulating effect of EA at ST25 on colonic motility has also been well demonstrated by our previous work (Sun et al., 2018a; Xu et al., 2019). PD has the characteristics of encephalo-intestinal disease and constipation is a precursor and one of its characteristic symptoms (Yu et al., 2018). The efficacy of EA at ST25 on PD motor function and constipation symptoms was confirmed by a randomized controlled multicentre trial (Li et al., 2023). In our previous study on the mechanism of EA at ST25 on PD constipation symptoms, its effects on motor symptoms were also observed (Song et al., 2022, 2023), and this paper further explored the therapeutic mechanism of motor symptoms on the basis of it. In the previous study, we also found that EA at ST25 changed the characteristics of gut microbiome in PD rats (Song et al., 2023). According to the current cutting-edge research progress, the changes of gut microbiome are closely related to the onset and treatment of PD (Travagli et al., 2020), which aroused our interest and prompted us to conduct related research. The present study firstly reappeared the effects of electroacupuncture (EA) on protecting motor function and dopaminergic neurons and modulation of gut microbial dysbiosis in rotenone-induced PD rat model. Then, possible pathways by which EA at ST25 influence PD via gut microbiome were explored through microbiota function prediction and fecal metabolomics changes.

## Materials and methods

### Establishment of the experimental animal model

In this study, eight-week-old Sprague Dawley (SD) rats were supplied by the Beijing Vital River Laboratory Animal Technology Co., Ltd. [No.110011220101889264, under grant SCXK(JING)2021–0011]. The experimental rats were kept in a barrier environment with stable parameters (conditions: 12/12 h light/dark cycle; temperature, 22 ± 2°C; relative humidity 60% ± 5%). The animals were randomly numbered and divided into three groups: The control group, the model group, and the EA group, with six animals in each group. They were kept in cages of the same size in groups with free access to food and water.

PD was induced by giving a low dose of rotenone (Cannon et al., 2009). Selection of the appropriate route of administration and concentration is critical for model stabilization and appropriate plasma concentrations (to avoid systemic toxicity) (Innos and Hickey, 2021). We formulated the following program based on previous research (Liu et al., 2015) and our prior investigations. The model and EA groups were injected subcutaneously with rotenone solvent on the back of the neck at a dose of 1 mL/kg once a day for 5 days a week. The solvent was prepared by dissolving 200 mg of rotenone (M6209; Abmole Bioscience Inc., Houston, TX, USA) in 3 ml of dimethyl sulfoxide (DMSO, D8370; Beijing Solarbio Science & Technology, Tongzhou, Beijing, China), and then fixed to 100 ml with sunflower oil to make up 2 mg/ml of rotenone sunflower oil solvent. The control group was injected with an equal volume of solvent mixture (3% DMSO sunflower oil solvent). Motor function was assessed at the end of each weekly intervention (refer to the “Motor coordination assessment” below for specific methods). The modeling was considered successful when there was a statistically significant difference in motor scores between the modeled rats and the control rats. This animal study was reviewed and approved by the Scientific Investigation Board of the Nanjing University of Traditional Chinese Medicine, Nanjing, China (permission no. 202112A047).

### Motor coordination assessment

The pole test and rotarod test (Park et al., 2017; Yan et al., 2022) were used to evaluate Motor mediation and balance impairment. The homemade climbing pole (100 cm high, 1 cm in diameter) was wrapped with medical gauze to ensure sufficient friction. The rats were placed on the top of the pole with their heads facing upward, and the time required to turn from vertical upward to vertical downward and to climb from the top of the pole to the bottom of the pole was recorded. The rotarod test used the KW-6D rat rod instrument (Nanjing Calvin Biotechnology Co., Ltd., Nanjing, Jiangsu, China). The total experiment time was 180 s. The first 90 s from 0 to 30 RPM uniform acceleration, the next 90 s maintain a uniform speed of 30 s per minute. Before the test, each rat was guided to perform two times of acclimatization training. Each rat was tested three times, with each interval of not less than 10 min, and the average value was taken.

### EA intervention

**Figure 1:**
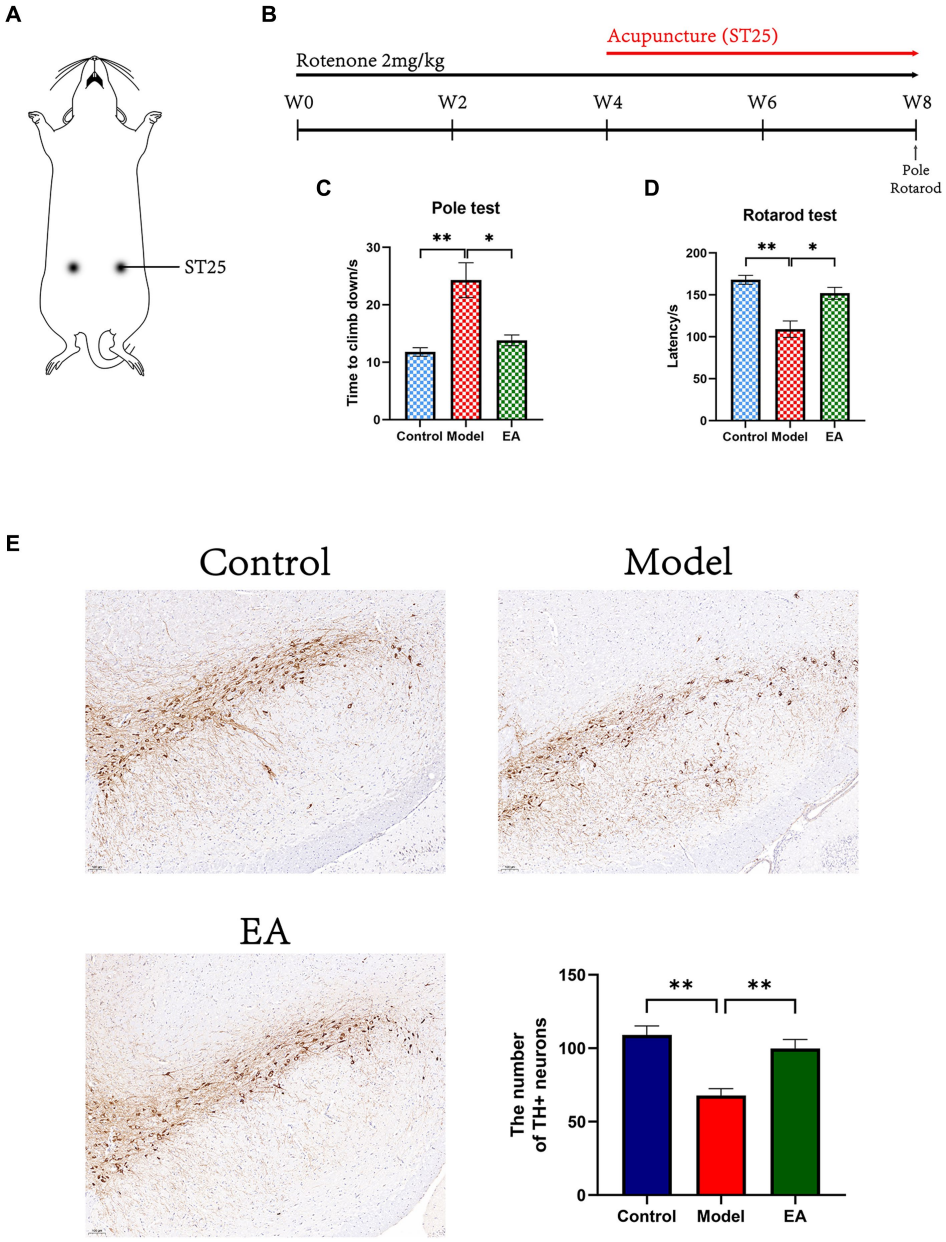
Effects of EA on motor functions in rotenone-induced PD rat. **(A)** EA on ST25 of PD rat treated with rotenone for 4 weeks (EA group). **(B)** Experimental schedule of the EA treatment in the rotenone-induced PD rats. All motor behavioral tests including **(C)** pole, and **(D)** rotarod tests were performed the ending day of the EA intervention (*n* = 6, Student *t*-test, ^*^ p < 0. 05, ^**^ p < 0. 01). **(E)** Immunohistostaining for tyrosine hydroxylase (TH) in the SN of brain extracted from Control, Model, and EA group and counts of TH^+^ neurons (*n* = 4. Scale bar: 100 μm). W, week.

The rats in the EA group were received EA treatment on bilateral ST25 (Tianshu, located 5 mm lateral to the intersection between the upper 2/3rd and the lower 1/3rd in the line joining the xiphoid process and the upper border of the pubic symphysis) after gas anesthesia with isoflurane (2–5%; 9020000522; Shenzhen Ruiwode Lift Technology). Meanwhile, the same anesthesia was administered to rats in the Model group but without performing EA. For the EA group, two stainless steel acupuncture needles (20162270970; Suzhou HUATUO Medical Instruments, Suzhou, Jiangsu, China) of 0.2 mm in diameter were inserted at a depth of 5 mm into the bilateral ST25 acupoint (Figure 1A). EA at ST25 was conducted with the HANS-100A (HAN ACUTENS WQ1002F; Beijing Anlong Photoelectric Technology, Haidian, Beijing, China) apparatus set to a current of 2 mA and a frequency of 2/15 Hz. EA treatment starts from the fifth week: 20 min a day for 5 days a week, 1 week a course, over four continuous courses of treatment.

### Samples collection

Samples were collected after 4 weeks of intervention. Stool samples were obtained using sterile instruments and containers and stored at –80°C. Blood samples were obtained by orbital blood sampling after anesthesia (Ethyl carbamate, 1,000 mg/kg, I.p), centrifuged (4°C, 3000 rpm, 15 min) within 1 h and the supernatant was stored at –80°C. After animals sacrificed under an overdose of anesthesia, fresh intact brains were pre-frozen in liquid nitrogen after removal, and subsequent treatments were carried out at low temperatures to ensure fixed tissue morphology and facilitate accurate sampling.

### DNA isolation and 16S rRNA gene sequencing

Total genome DNA from samples was extracted using the CTAB method. DNA concentration and purity was monitored on 1% agarose gels. 16S rRNA genes of distinct regions (16S V3-V4) were amplified used specific primers 338F (ACTCCTACGGGAGGCAGCAG) and 806R (GGACTACHVGGGTWTCTAAT) with the barcode. PCR products were purified with Qiagen Gel Extraction Kit (Qiagen, Germany). Sequencing libraries were generated using TruSeq® DNA PCR-Free Sample Preparation Kit (Illumina, USA) following the manufacturer’s recommendations and index codes were added. The library quality was assessed on the Qubit@2.0 Fluorometer (Thermo Scientific) and Agilent Bioanalyzer 2,100 system.

The library was sequenced on an Illumina NovaSeq platform, generating 250 bp paired-end reads. First, the raw data were screened and sequences were removed if they were shorter than 200 bp, had a low-quality score (≤20), contained ambiguous bases or did not match primer sequences and barcode tags. Qualified reads were separated based on the sample-specific barcode sequences and trimmed with Illumina Analysis Pipeline Version 2.6. Then, the dataset was analyzed using VSEARCH. The sequences were clustered into operational taxonomic units (OTUs) at a similarity level of 97%, to generate rarefaction curves and calculate the richness and diversity indices. The Ribosomal Database Project (RDP) Classifier tool was applied to classify all sequences into different taxonomic groups. Clustering analyses and principal component analysis (PCA) were used based on OTU information from each sample using R-Studio to examine the similarity between different samples.

linear discriminant analysis (LDA) Effect Size (LEfSe) analysis was used to find species that differed significantly in abundance between groups (i.e., biomarkers). Species with significant differences in abundance between groups were first detected using the parametric test ANOVA test in multiple samples, with a threshold set at 0.05. Then the significantly different species obtained in the previous step were analyzed for between-group differences using the Wilcoxon rank sum test in groups, with a threshold set at 0.05. Finally, LDA was used to downscale the data to assess the significant differences in the influence of species (i.e., LDA score) was assessed using LDA, with a threshold set of 3/3.5. The obtained differential species were used for the next analysis.

### Metabonomic analysis based on liquid chromatography-mass spectrometry

The sample stored at-80°C refrigerator was thawed on ice. A 400 μl solution (Methanol: Water = 7:3, V/V) containing internal standard was added into 20 mg sample, and vortexed for 3 min. The sample was sonicated in an ice bath for 10 min and vortexed for 1 min and then placed at –20°C for 30 min. The sample was then centrifuged at 12,000 rpm for 10 min (4°C). And the sediment was removed, then centrifuged the supernatant was at 12,000 rpm for 3 min (4°C). A 200 μl aliquots of supernatant were transferred for LC–MS analysis.

All samples were acquired by the LC–MS system following machine orders. The analytical conditions were as follows, UPLC: column, Waters ACQUITY UPLC HSS T3 C18 (1.8 μm, 2.1 mm*100 mm); column temperature, 40°C; flow rate, 0.4 ml/min; injection volume, 2 μl; solvent system, water (0.1% formic acid): acetonitrile (0.1% formic acid); The column was eluted with 5% mobile phase B (0.1% formic acid in acetonitrile) at 0 min followed by a linear gradient to 90% mobile phase B (0.1% formic acid in acetonitrile) over 11 min, held for 1 min, and then come back to 5% mobile phase B within 0.1 min, held for 1.9 min.

The original data file acquired by LC–MS was converted into mzML format by ProteoWizard software. Peak extraction, peak alignment, and retention time correction were, respectively, performed by the XCMS program. The “SVR” method was used to correct the peak area. The peaks with a detection rate lower than 50% in each group of samples were discarded. After that, metabolic identification information was obtained by searching the laboratory’s self-built database, integrated public database, AI database, and metDNA.

Unsupervised PCA was performed by statistics function prcomp within R. The data was unit variance scaled before unsupervised PCA. For two-group analysis, differential metabolites were determined by VIP (VIP > 1) and *p*-value (p-value <0.05, Student’s *t*-test). VIP values were extracted from OPLS-DA results and were generated using the R package MetaboAnalystR. The data was log transform (log2) and mean centering before orthogonal partial least squares discriminant analysis (OPLS-DA). In order to avoid overfitting, a permutation test (200 permutations) was performed. Identified metabolites were annotated using the KEGG Compound database,^1^ and annotated metabolites were then mapped to the KEGG Pathway database.^2^ Significantly enriched pathways are identified with a hypergeometric test’s *p*-value for a given list of metabolites.

### Lipidomics analysis of serum and brain samples

The lipids of individual serum or brain SNPC samples were extracted by using a modified protocol of Bligh and Dyer (1959) in the presence of internal standards as previously described (Han and Gross, 2005). Each lipid extract was resuspended with 200 μl chloroform/methanol (1:1, v/v)/mg protein, and stored at –20°C for lipid analysis. Derivatization of the primary amine in phosphoethanolamine-containing species (such as PE and lysoPE) with fluorenyl methoxycarbonyl chloride was conducted according to the previously reported method (Han, 2005; Hu et al., 2020). Individual lipid species including FA isomers and regioisomers were identified using multidimensional MS analysis (Yang et al., 2009; Hu et al., 2022).

Lipidomics analysis of serum and brain samples was conducted with a triple-quadrupole mass spectrometer (Thermo TSQ Quantiva) equipped with an automated nanospray ion source (TriVersa NanoMate, Advion Bioscience Ltd., Ithaca, NY, USA) as previously reported (Han and Gross, 2005). To prevent possible lipid aggregation, the solutions of lipid extracts were diluted in CHCl_3_/MeOH/isopropyl alcohol (1:2:4, v/v/v) prior to direct infusion. All mass spectral data were acquired by different customized sequence subroutines operated under Xcalibur software (Xcalibur 3.0, Thermo Fisher Scientific Inc., San Jose, CA, USA). Data processing was performed based on the previous method (Yang et al., 2009).

### Analysis of serum cytokines and endotoxin

The serum concentrations of interleukin-6 (IL-6), IL-10, tumor necrosis factor-alpha (TNF-α), interferon-gamma (IFN-γ), and lipopolysaccharide (LPS) were determined using ELISA assay kits (Quanzhou Ruixin Biological Technology Co., Ltd., Meile Street East Section, Dacheng Town, Sanyuan County, Shaanxi. China) according to the manufacturer’s protocol.

### Data analysis

All data analyses were performed using GraphPad Prism 9.4 (GraphPad Inc., La Holla, CA, USA) and R. *p* < 0.05 was considered to indicate statistical significance. As a variety of statistical methods are used, specific methods will be marked in detail in the Methods and Results section and figure note.

## Results

### EA improves motor dysfunctions in rotenone-induced PD rats

Experimental schedule of the EA treatment in the rotenone-induced PD rats was shown in Figure 1B. At the end of the 4-week rotenone intervention, the modeled rats showed a significant decrease in motor function (Supplementary Figure S1). EA intervention was started from week 5 and after 4 weeks of intervention, the motor coordination ability of rats in each group was compared. In the pole test, the time climbing down of rats in the Model group were significantly extended (*p* < 0.01 vs. Control group), from about 10s in the Control group to more than the 20s in the Model group, and the change factor was more than two times (Figure 1C). The time climbing down of the EA group was significantly reduced compared to the Model group (*p* < 0.05). In the rotarod test, the reduction of latency time induced by rotenone injection (*p* < 0.001 vs. Control group) was restored by the EA treatment (*p* < 0.001 vs. Model group; Figure 1D).

### EA counteracts rotenone-induced deficits in dopaminergic neurons

We confirmed the neuroprotective effects of EA on dopaminergic neurons in the SNpc of rotenone-induced rats. In the SN, a significant difference was observed among the three groups in the number of TH-positive neurons. There were decreased in the Model group (*p* < 0.01 vs. Control group) and were increased in the EA group (*p* < 0.01 vs. Model group; Figure 1E).

### EA alters dysbiosis of the gut microbiome in rotenone-induced PD rats

The dilution curve (Rarefaction curve), Shannon-Wiener curve, species accumulation curve, and goods coverage index generated by OTUs indicate that all samples achieved high sampling coverage (≥99%) (Supplementary Figure S2; Supplementary Table S1). This indicates that the sequencing depth is sufficient for studies of the gut microbiota. The Venn diagram shows 1,000 unique OTU in the control group,1,575 unique OUTs in the model group, and 1,012 unique OUTs in the EA group. There was 1,118 OTU in the EA group but not in the Model group (Supplementary Figure S3A).

**Figure 2:**
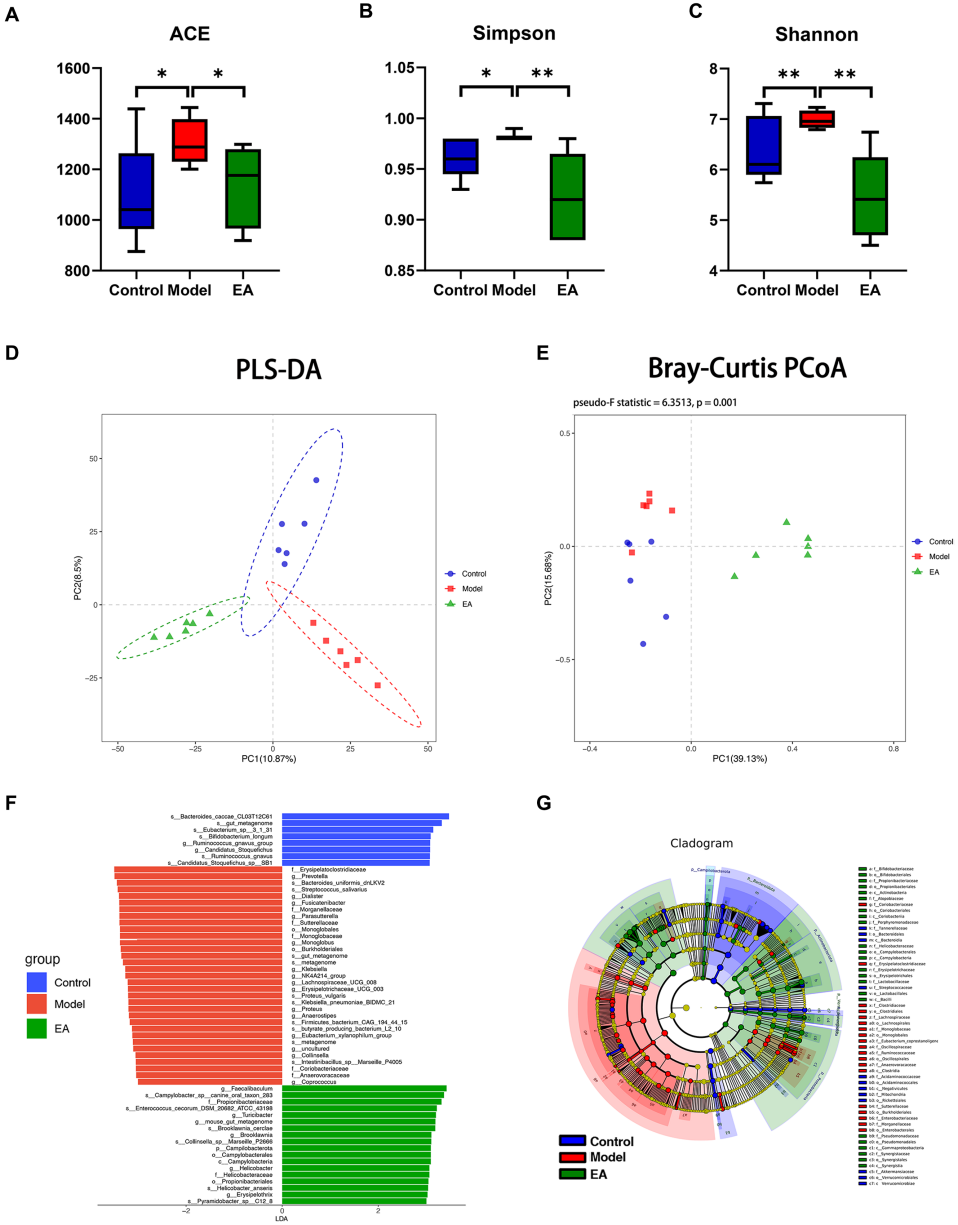
Diversity analysis of gut microbiota. Rotenone treatment increased ACE **(A)**, Simpson index **(B),** and Shannon **(C)** of the model group (*n* = 6, Student *t*-test, ^*^ p < 0.05, ^**^ p < 0.01). Compared with the control group, EA decreased ACE, Simpson index and Shannon index **(A–C)**. PLS-DA and PCoA (PERMANOVA: Pseudo-F Statistic = 6.3513, *p* = 0.001) analysis showed that the bacterial communities in each group were far apart and clearly demarcated **(D,E)** (Amova: *p* < 0.001; Anosim: *R* = 0. 9,593, *p* < 0. 01). **(F)** LDA scores for the bacterial taxa differential abundant (LDA > 3.5). **(G)** Cladograms generated by LEfSe indicate differences in the bacterial taxa (LDA > 3.0).

It was found by α-diversity analysis, rotenone treatment increased the community richness (Figure 2A; Supplementary Figures S3B,C) and diversity (Figures 2B,C) of intestinal microbiota, suggesting that the model group had gut microbiota disorder, and EA intervention played a positive role in this situation. β-diversity analysis showed that there were significant differences among the three groups (Figures 2D,E; Supplementary Figures S3D,E), the model group and control group separated along the longitudinal axis, the EA group and model group separated along the horizontal axis, partial least squares discriminant analysis (PLS-DA) showed the most obvious difference (Figure 2D). The LDA distribution diagram analysis (LAD score > 3.5) showed the differential microbiota in model group rats, including 2 orders, 6 families, 15 genera, and 10 species (Figure 2F). After the EA intervention, 1 phylum, 1 class, 2 orders, 2 families, 6 genera, and 6 species were significantly altered (Figure 2F). The cladogram shows the differential microbiota after the LAD score threshold was relaxed to 3.0 (Figure 2G).

**Figure 3:**
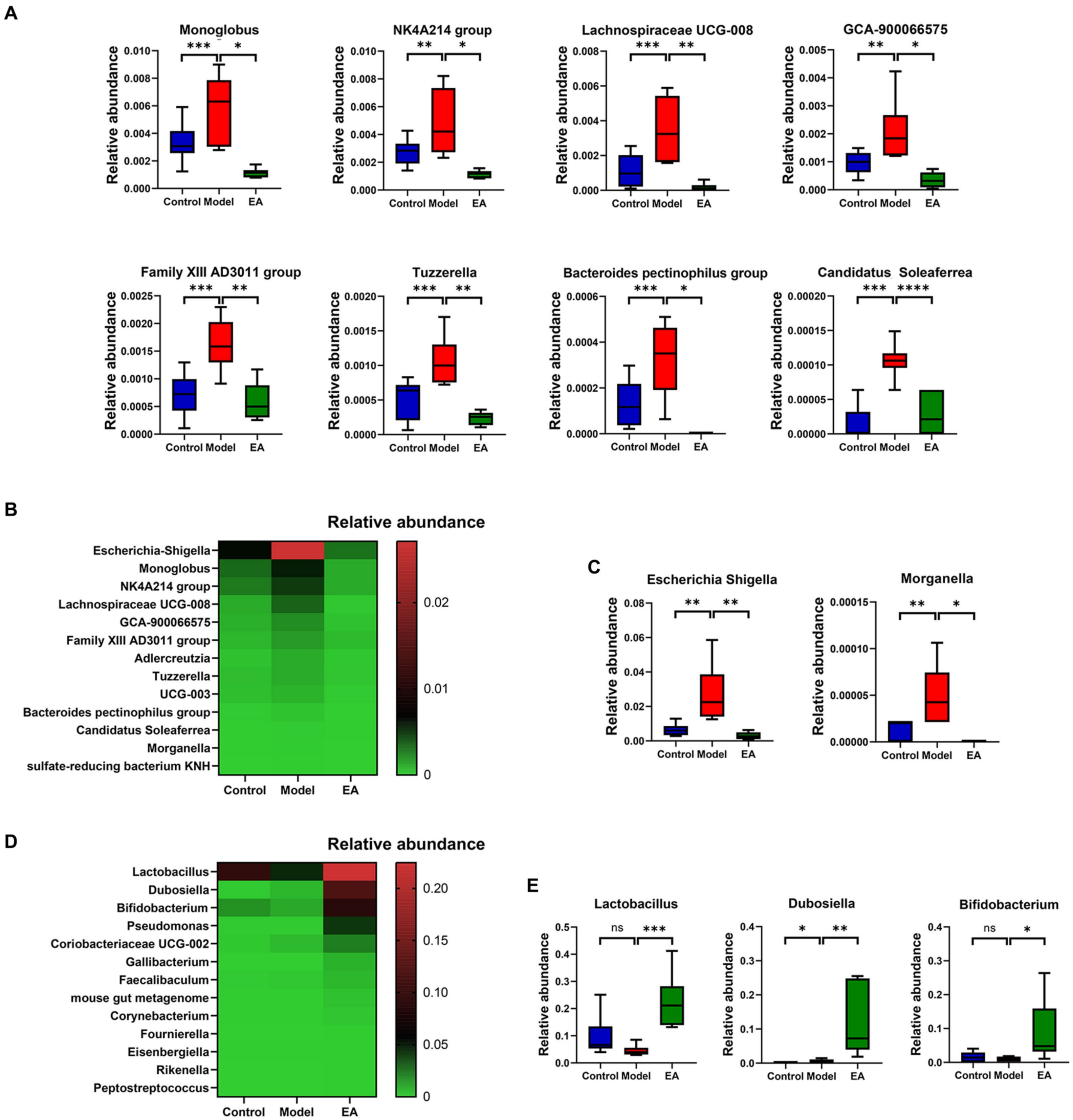
Analysis of species differing at the genus level. **(A)** Differential microbiota at genus level belong to *Bacillota*. **(B)** Genera whose relative abundance was significantly increased in the Model group and decreased in the EA group, **(C)** of which *Escherichia-Shigella* and *Morganella* belong to *Pseudomonadota*. **(D)** Genera whose relative abundance was significantly increased in the EA group, **(E)** of which relative abundance of *Lactobacillus*, *Dubosiella,* and *Bifidobacterium* in the EA group was >0.05. (*n* = 6, Metastats, ^*^p < 0.05, ^**^p < 0.01, ^***^p < 0.001, ^****^p < 0.0001).

Genus-level analyses showed a significant distinction between the gut microbiota of the EA and Model groups. A total of 68 genera were identified and differed significantly among the three groups (Supplementary Table S2; FDR *p* < 0.05, Kruskal–Wallis one-way ANOVA). Among these genera, 15 were significantly different between the Control and Model groups (Supplementary Table S2; *p* < 0.05, Metastats); 14 genera of them in the Model group were enriched compared to the Control group. The EA treatment significantly reversed the changes in the relative abundance of 13 genera (Figure 3B), of which *Escherichia-Shigella* and *Morganella* belonged to *Pseudomonadota* (Figure 3C). Among these 13 genera, the relative abundance of *Escherichia–Shigella* was far ahead. In addition, compared to the Model group, 13 genera were significantly elevated in the EA group (Figure 3D), especially *Lactobacillus*, *Dubosiella,* and *Bifidobacterium* (Figure 3E).

**Figure 4:**
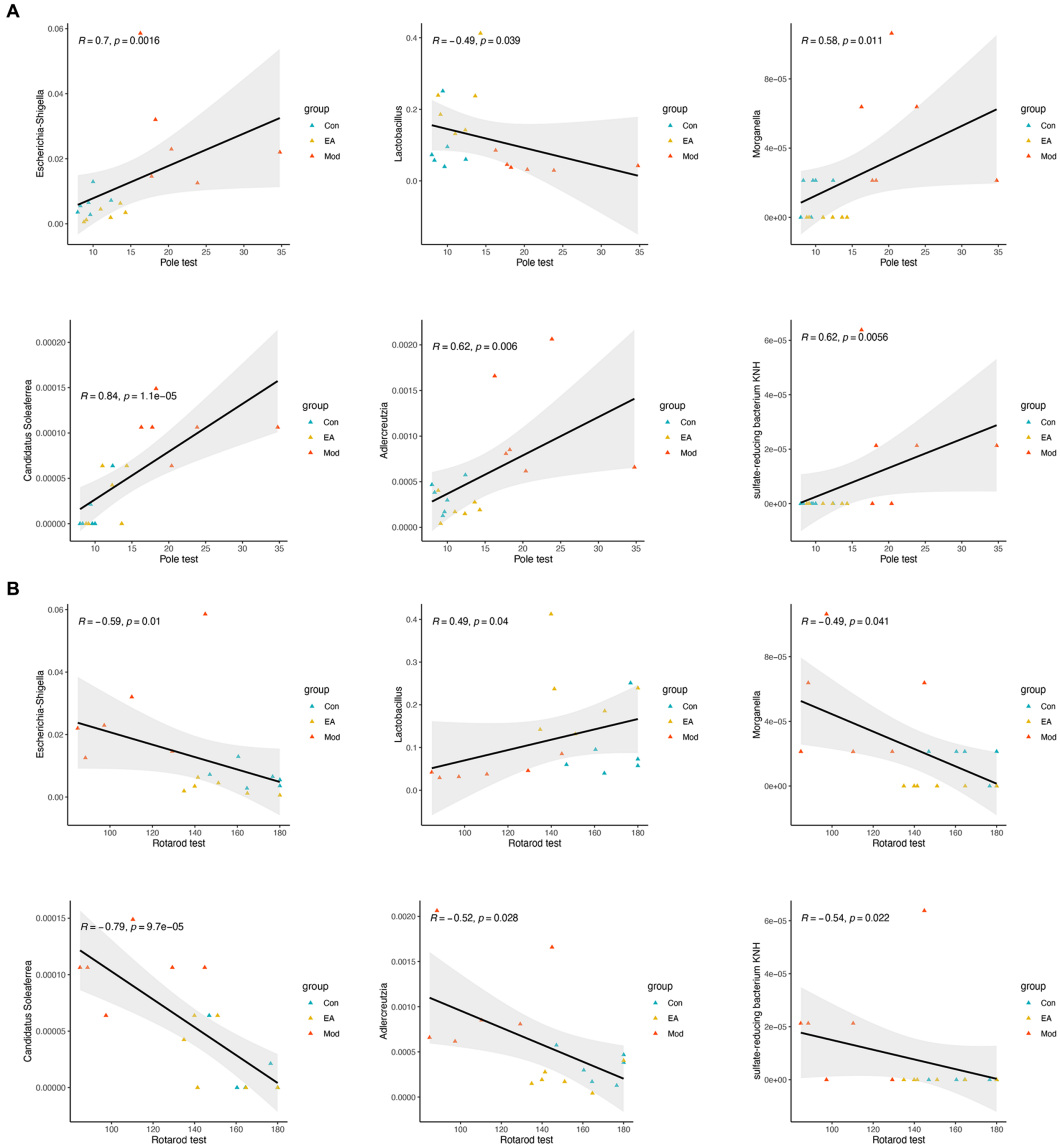
Correlation between the bacteria of genus and the behavior functions. Spearman’s correlation analyses between the relative abundance of gut microbiota at the genus level and the value in **(A)** pole or **(B)** rotarod test, respectively. Significant correlations were determined based on |Spearman *r*| > 0.5 and *p* < 0.05.

To examine whether the gut microbiota correlates with PD clinical features, and also to determine whether the extensive changes in the gut microbiota and clinical features of PD following EA treatment are associated, we further analyzed the relationship between the gut microbiota and motor functions using Spearman’s correlation, based on above 68 genera (Supplementary Table S2) and the representative parameters for behavioral deficits in the Model group rats, the pole and rotarod test. The correlation analyses indicated that 11 and 6 genera were significantly correlated with the pole and rotarod test, respectively (Supplementary Table S3). Interestingly, *Lactobacillus*, *Escherichia-Shigella*, *Morganella*, *Adlercreutzia*, *Candidatus Soleaferrea*, *sulfate-reducing bacterium KNH* were correlated with the rotarod and pole test (Figure 4).

### EA changes the functionality of the gut microbiota in rotenone-induced PD rats

**Figure 5:**
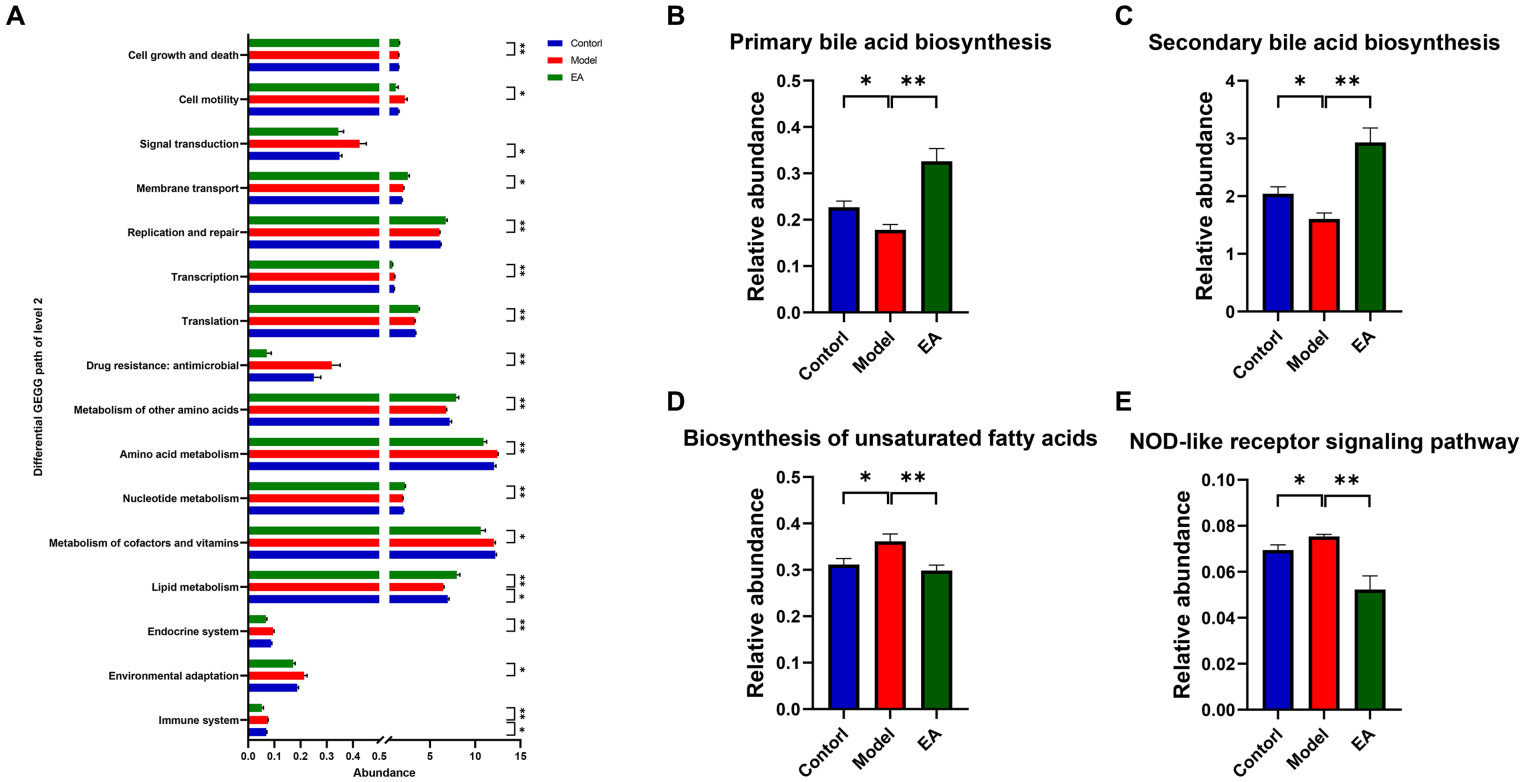
Microbial gene functions annotation on KEGG. **(A)** Differential KEGG path of level 2. **(B–E)** The relative abundance of the predicted KEGG pathways among the Control, Model, and EA groups. (*n* = 6, Wilcoxon rank sum test, ^*^p < 0.05, ^**^ p < 0.01).

To gain insight into the functional categories and pathways associated with EA treatment in PD rats, we used PICRUSt2 to compare the function of gut microbiota between the three groups. A total of 89 KEGG pathways at level 3 were significantly altered in the Model or EA group (*p* < 0.05), and many of the predicted functional differences were in metabolic pathways (Supplementary Table S4). Based on the grade 2 KEGG pathway findings, the immune system, lipid metabolism, and signal transduction were disturbed in the model group of rats. EA affected 16 signaling pathways, 5 of which belonged to metabolic pathways, occupying 1/3 of the total proportion. The immune system and lipid metabolism were both associated with the pathogenesis of PD and the intervention role of EA (Figure 5A). Therefore, we performed further analysis of immune system and lipid metabolism based on grade 3 KEGG pathways. The results show that 4 pathways, including Biosynthesis of unsaturated fatty acids, primary bile acid biosynthesis, secondary bile acid biosynthesis, and NOD-like receptor signaling pathway were disturbed in the model group, and EA affects 8 signaling pathways, among which Biosynthesis of unsaturated fatty acids, primary bile acid biosynthesis, secondary bile acid biosynthesis, and NOD-like receptor signaling pathway is related both to the pathogenesis of PD and the intervention effect of EA (Supplementary Table S4; Figures 5B–E).

### Fecal metabolomics analysis revealed that EA regulates abnormal metabolic patterns in PD rats

**Figure 6:**
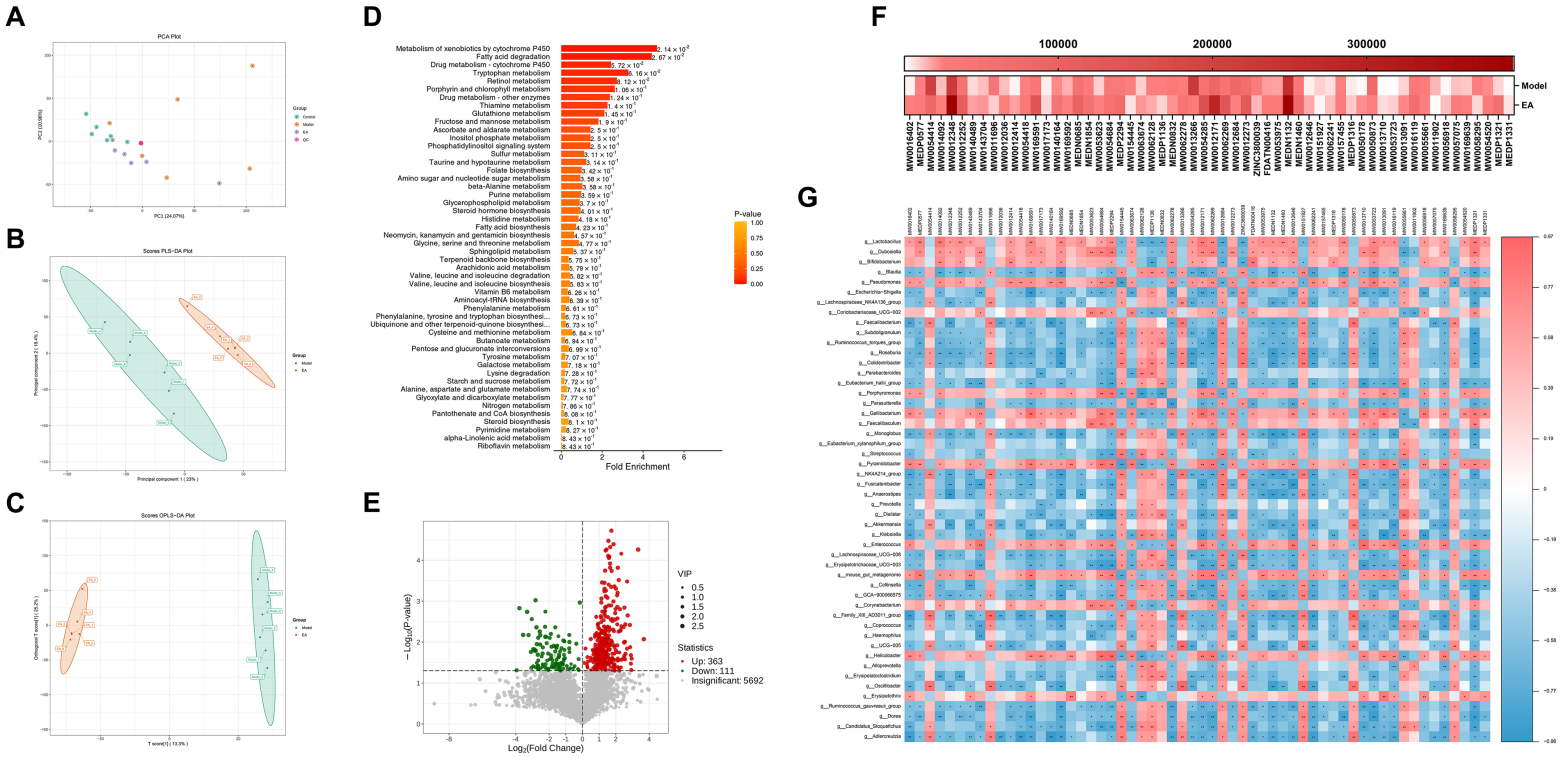
Fecal metabolomics analysis. **(A)** PCA of the control, model and EA group. **(B,C)** PLS-DA and OPLS-DA showed an obvious separation trend of fecal metabolites in the model and EA group. **(D)** The first 50 differential metabolisms were selected by MSEA enrichment analysis. **(E)** The volcanic map shows different metabolites between the EA and Model groups. (F) 58 lipid components among the differential metabolites. **(G)** Correlations between fecal microbiota and metabolites.

Microbial-derived metabolites affect the host through multiple pathways. Increasing evidence suggests that some metabolites of the gut microbiota can enter the bloodstream, with important effects on the physiology and behavior of the host (Sarkar et al., 2016; Martin et al., 2018). Next, we investigated the host metabolic profile in the same samples as the 16S rRNA analysis by liquid chromatography-mass spectrometry (LC/MS) and explored the relationship between microbiota and metabolites. Principal component analysis showed that the fecal samples of rats from the model group and the control group were significantly separated along the horizontal axis. The model group is more separated within the group than the control and EA groups and does not form well clusters (Figure 6A). According to the PLS-DA and the orthogonal partial least squares discriminant analysis (OPLS-DA) (Figures 6B,C), there was a large degree of separation between the EA and model groups, indicating different metabolic patterns. There are 57 metabolic sets that were significantly different between the two groups (Figure 6D), including 17 lipid metabolic pathways such as butyrate metabolism, fatty acid biosynthesis and degradation, and glycerophospholipid metabolism.

Screening the differential metabolites between EA and model groups by VIP > 1 and *p* < 0.05, a total of 474 differential metabolites were obtained, of which 363 increased and 111 decreased compared with the model group (Figure 6E). Fold Change (FC) values were performed and 92 metabolites changed 2-fold and 10 metabolites changed 3-fold (Figure 6E; Supplementary Table S2). Secondary screening of lipid components among the differential metabolites was performed, and a total of 58 differential lipids were obtained, of which 44 increased and 14 decreased compared to the model group (Figure 6F), with most lipid metabolites showing an elevated state. To explore the potential relationship between gut microbiota changes and metabolites, a correlation matrix was generated using Spearman correlation (Figure 6G). Most of the lipids were negatively correlated with *Escherichia–Shigella* and positively correlated with *Lactobacillus*, *Dubosiella,* and *Bifidobacterium*, with the exception of a few such as PA (18:0/16:0) and PE [18:2(9Z,12Z)/20:2(11Z,14Z)].

### EA reversed the lipid peroxidation of SNpc by regulating plasmalogen

**Figure 7:**
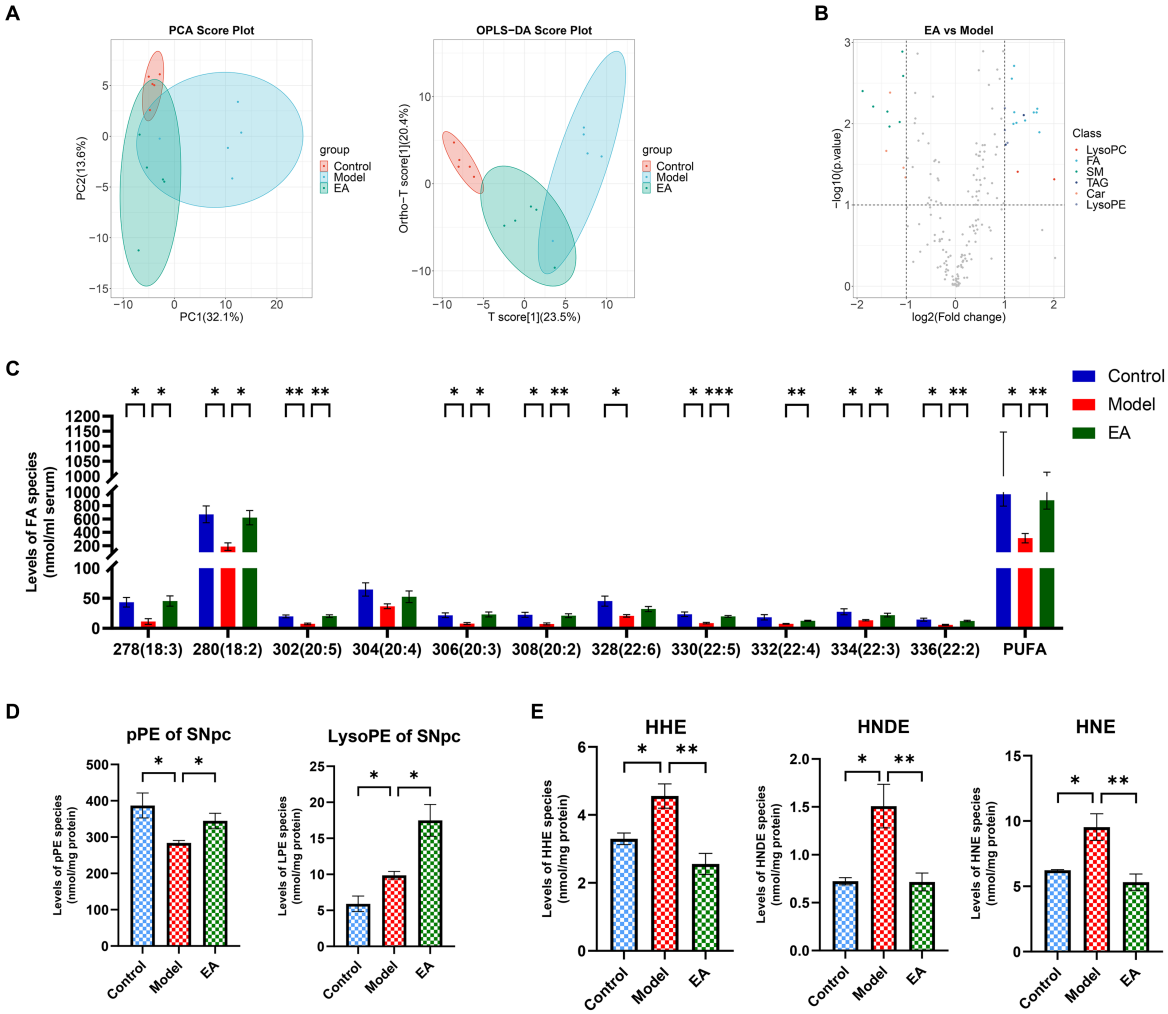
Representative comparison of lipidomics data of serum and SNpc. **(A)** PCA and OPLS-DA analysis of serum in the control, model and EA group. **(B)** The volcanic map shows different serum lipid metabolites between the EA and Model groups. Lipidomics analysis of **(C)** polyunsaturated fatty acids (PUFAs) of serum, **(D)** plasmalogen PE (i.e., plasmenylethanolamine, pPE) and ethanolamine lysoglycerophospholipid (LPE) of SNpc, and (E)4-hydroxyalkenal species of SNpc presented in lipid extracts was conducted by using multi-dimensional mass spectrometry-based shotgun lipidomics (*n* = 5, Student *t*-test, *p < 0.05, **p < 0.01, ***p < 0.001). The prefix “p” in **(D)** stands for plasmalogen PE species which consist of a vinyl ether linkage of the aliphatic chain at the sn-1 position. **(E)** HHN, HNDE, and HNE denote 4-hydroxy-2(E)-hexenal, 4-hydroxy-2(E)-nondinenal, and 4-hydroxy-2(E)-nonenal, respectively.

Based on the above results, we focused on abnormal lipid metabolism. Serum lipidomics analysis indicated that the serum lipids of the Control group and the Model group showed completely different characteristics. Specifically, the two groups showed obvious separation trend along the horizontal axis, and the Model group did not form a good clustering., while EA treatment could partially reverse this trend (Figure 7A). The volcano map showed that the lipid type with the highest proportion and the most significant difference in metabolites between the Model group and the EA group was FA (Figure 7B). Further analysis revealed that rotenone intervention reduced the concentration of almost all detected PUFA types (Figure 7C) which was in line with the prediction of microbial function above (Figure 5D), and EA treatment reversed this change. In addition, changes in lysophospholipids also attracted our attention (Figures 7B,D), which may be attributed to the significantly reduced serum PUFA content. Compared with the Control group, plasmenylethanolamine (pPE) content in the Model group was significantly increased, while ethanolamine lysoglycerophospholipid (LPE) content showed an increasing trend without statistical difference (this portion of the data is not displayed here). This indicates that under oxidative stress, the body generated more plasmalogen species to prevent increased oxidative stress, and thereby lipid peroxidation.

This did not seem to be consistent with the basic pathological features of PD, so we further examined pPE and LPE levels in SNpc. Compared with Control group, the content of pPE in SNpc in Model group was significantly reduced, while the content of LPE was significantly increased. The EA intervention increased both pPE and LPE levels (Figure 7D). We hypothesized that this trend was due to the presence of lipid peroxidation, for oxidative stress was already present in SNpc, and that the EA intervention acted as an antioxidant by increasing phospholipid concentrations. To verify this hypothesis, we detected lipid peroxidation products in SNpc. The results showed that the contents of 4-hydroxy-2(E)-hexenal (HHN), 4-hydroxy-2(E)-nondinenal (HNDE), and 4-hydroxy-2(E)-nonenal (HNE) in SNpc in Model group were significantly increased, and EA treatment reduced their contents (Figure 7E), which verifies our conjecture above. The different trends of pPE and LPE in SNpc and serum suggest that the lack of antioxidant capacity in SNpc may be the reason for the preferential damage of dopaminergic neurons in the oxidative stress state compared with the systemic state.

### Endotoxin-mediated systemic inflammation associates gut microbiota and dopamine neuron damage

**Figure 8:**
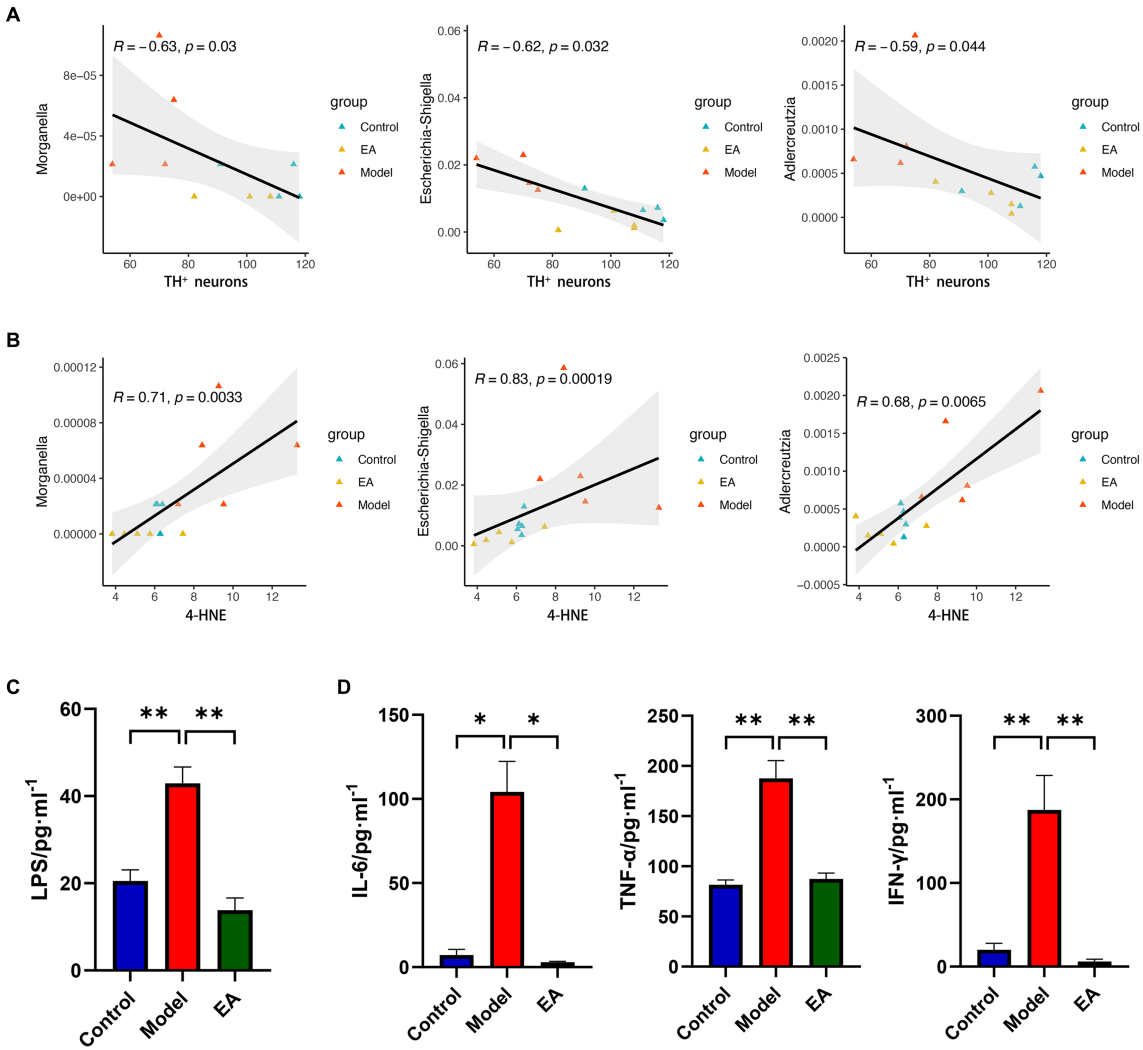
Bacterial endotoxin links lipid peroxidation with TH+ neuronal damage. Spearman’s correlation analyses between the relative abundance of gut microbiota at the genus level and the value of **(A)** TH^+^ neurons or **(B)** 4-HNE, respectively. Significant correlations were determined based on |Spearman *r*| > 0.5 and *p* < 0.05. Serum LPS **(C)** and inflammatory responses **(D)** in each group (*n* = 3–4, Student *t*-test, ^*^p < 0. 05, ^**^p < 0. 01).

To further determine the role that gut microbiota plays in EA regulation of lipid peroxidation in SNpc, we analyzed the relationship between the 68 screened genera (Supplementary Table S2) and TH^+^ neurons or the lipid peroxidation product, 4-hydroxyalkenals (4-HNE), in SNpc, respectively. The correlation analyses indicated that 4 and 34 genera were significantly correlated with TH^+^ neurons and 4-HNE, respectively (Supplementary Table S6). Three genera, *Morganella*, *Escherichia-Shigella*, and *Adlercreutzia*, two of which belong to the *Pseudomonadota* (previously known as *Proteobacteria*), were correlated with both TH^+^ neurons (Figure 8A) and 4-HNE (Figure 8B), which positively correlated with 4-HNE and negatively with TH^+^ neurons. The inflammatory response caused by the release of LPS from Gram-negative bacteria has long been constituted as an important etiologic factor in PD, and different routes and modes of LPS administration can cause various parkinsonian symptoms (Jia et al., 2023). The up-regulation of NOD-like receptor signaling pathways (Figure 5E) in the Model group also suggests the presence of LPS-mediated inflammatory cascades. Then we tested serum LPS and inflammatory factors. Rats in the Model group showed generalized elevated LPS (Figure 8C) and inflammatory responses (Figure 8D) and EA reversed this change.

## Discussion

Gut microbial dysbiosis and alteration of microbial metabolites are involved in the development of PD (Sun et al., 2018b). Dysbiosis in the composition and abundance of gut microbiota can affect both the enteric nervous system (ENS) and the CNS in PD pathogenesis. Substantial evidence supported the functional effects of microbiota on the bi-directional communications between gut and brain, indicating the existence of a microbiome-gut-brain axis that induces neuroinflammation and deficiency of dopaminergic neurons and thereby leading to PD (Wang et al., 2021). Alterations in the diversity and composition of gut microbiota and microbial products are found both in PD patients and PD-related animal models. Previous study has shown that acupuncture can induce normalization of gut microbial dysbiosis as well as inhibition of neuroinflammation in the SN and the ST, which are associated with PD phenotypes (Jang et al., 2020). However, the intermediate link between regulation of intestinal microbiome disorder and improvement of pathological changes of CNS by acupuncture is still unclear.

### Gut microbiology studies uncover that improvement of PD motor symptoms is tied to microbiota recovery

Acupuncture has been considered a potential nondrug therapy for PD patients. Growing evidences showed that acupuncture has been used to improve brain functions and inflammation in neurological disorders such as PD (Tamtaji et al., 2019a), and also to recover the gastrointestinal dysfunctions in various metabolic disorders (Si et al., 2018). Similarly, these studies suggest that gut microbiota may be a target for acupuncture treatment, thereby playing a crucial role in mutual regulation of the brain-gut axis (Wei et al., 2019). In the recent study, acupuncture improves PD symptoms and regulates gut microbial composition in 1-methyl-4-phenyl-1,2,3,6-tetrahydropyridine (MPTP)-induced PD mice model (Jang et al., 2020). Another study reported that EA may alleviate behavioral defects via modulation of gut microbiota and suppression of inflammation in the SNpc of mice with PD (Han et al., 2021).

In this study, the β-diversity analysis emerges a significant difference in the gut microbiota (Figures 2D,E). There was a little overlap in the distribution of the Model and Control groups. EA-treated rats formed a unique cluster that was separate from the control and PD rats, indicating that the EA treatment completely altered the composition of the gut microbiota, which is also well corroborated by the change in genus level (Supplementary Table S2; Figure 3). The EA intervention significantly altered 68 genera, much more than the 15 genera treated with rotenone. In addition to reversing changes in genus levels in the vast majority of the model groups, most of the alterations appeared to be specific to the EA treatment. In terms of α-diversity analysis, the richness (Figure 2A) and diversity (Figures 2B,C) of gut microbiota in rotenone-induced PD rats were significantly altered, that ACE, Shannon and Simpson indices were significantly increased compared with those in the Control group (For the Simpson Index, we used Simpson’s Index of Diversity 1-D to deal with it intuitively). EA reversed the change of microbiota richness and diversity index in Model group. This finding does not fit with the conventional wisdom that the richness and diversity of gut microbiota tend to decline during disease states. However, among all the PD patient gut microbiota studies that reported significant differences in alpha diversity, the vast majority (five out of six) had significantly increased gut microbiota richness (Chen et al., 2021). Similar animal results have been reported for changes in the gut microbiota of Parkinson’s disease (Cao et al., 2023). What led to the counterintuitive changes in model group diversity and richness, and what do the unique changes resulting from the EA treatments mean (are those 53 genera changes serving to adjust for gut microbiome disruption)? These need to be concluded by species-specific analyses.

At the phylum level, there is evidence that the *Pseudomonadota* (previously *Proteobacteria*) is a marker of dysbiosis in the gut microbiome, with pro-inflammatory characteristics, which generally overexpressed in gastrointestinal and immune diseases (Shin et al., 2015). *Pseudomonadota* contributes to the elevated luminal lipopolysaccharide (LPS) levels in PD, which may impair intestinal integrity (Nighot et al., 2017). LPS-triggered immune process can affect the CNS through the gut-brain axis, activating microglia and leading to dopaminergic neuron death (Deng et al., 2020). However, the abundance of *Pseudomonadota* did not change significantly at the phylum level, so we further looked at changes at the genus level. Preliminary analysis shows that *Bacillota* (previously known as *Firmicutes*) has the maximum number of species with significant differences between the Control and Model groups, not *Pseudomonadota* (Supplementary Table S2; Figure 3A). One step further, different genera with opposite trends in Model group and EA group were screened out, and the abundance of *Escherichia–Shigella*, which belonging to *Pseudomonadota*, was absolutely dominant (Figure 3B). *Morganella*, belongs to the same phylum, *Pseudomonadota*, was associated with major depression and genetic damage (Qin et al., 2022). Moreover, we showed that the abundances of the genera *Escherichia–Shigella*, *Lactobacillus,* and *Dubosiella* were highly correlated with behavioral metrics of motor deficits (both pole and rotarod tests) (Figure 4). Concerning motor symptoms, it has been observed that the pro-inflammatory taxa *Escherichia* and *Serratia* are also prevalent in non-tremor dominant patients with PD (Vascellari et al., 2021). Additionally, *Escherichia coli* and *Salmonella* produce bacterial amyloids (Yan et al., 2020), which positively promoted α-syn pathology in the gut and brain (Sampson et al., 2020). This is in line with our observation of higher *Escherichia–Shigella* abundance in PD rats.

On the other hand, the genera with significant increase in EA group were screened out, in which the relative abundance of *Lactobacillus*, *Dubosiella,* and *Bifidobacterium* exceeded 0.05. Reportedly, *Lactobacillus* is generally considered beneficial and often included in probiotic mixtures (Skrzydlo-Radomanska et al., 2020; Romano et al., 2021), which could represent a compensatory mechanism to restore intestinal homeostasis. Previous studies have shown that acupuncture can protect the gut microbiota by promoting the growth of *Lactobacillus* and inhibiting the proliferation of *Pseudomonadota* and *Escherichia-Shigella*, thereby exerting a therapeutic effect on many diseases such as PD (Han et al., 2021), Alzheimer’s disease (Zhang Y. et al., 2022), depression (Wang et al., 2020), and ulcerative colitis (Song et al., 2020). The studies of gut microbiota in PD appear complex and inconsistent, although the consistent or contradictory results compared with other studies (Vascellari et al., 2020). Short-chain fatty acids (SCFA) and their production bacteria were found to be generally reduced in the intestines of PD patients, which may interact with increased intestinal mucosal permeability and endotoxin exposure (Yang et al., 2019). *Dubosiella* and its metabolite butyrate protect neurons from LPS-induced damage by inhibiting microglia-mediated inflammation and oxidative stress through the GPR109A/Nrf2/HO-1 pathway (Zhang H. et al., 2022). Although there was no difference in the relative abundance of *Bifidobacterium* among the three groups (Kruskal–Wallis one-way ANOVA), considering that this difference may only appear in EA (according to Metastats, *Bifidobacterium* has a significant difference between EA group and Model group) and its own special role, *Bifidobacterium* has also been included in the differential genus. Both clinical and cellular experiments have found that *Lactobacillus* and *Bifidobacterium* can reduce inflammation and oxidative stress and improve clinical symptoms in patients with Parkinson’s disease (Magistrelli et al., 2019; Tamtaji et al., 2019b). One investigation showed that *Bifidobacteria* showed a significant advantage after mosapride intervention, which may be related to increased intestinal motility and reduced plasma endotoxin levels with mosapride (Chen et al., 2022). EA at ST25 has also been shown to improve intestinal peristaltic capacity and ENS function (Song et al., 2022). Combined with our results on microbiome analyses, it can be hypothesized that EA may inhibit neuroinflammation, protect DA neurons and improve motor impairment in PD rats by improving the relative abundance of gut microbiota, such as inhibition of *Escherichia–Shigella* by increasing the abundance of *Lactobacillus*, *Dubosiella,* and *Bifidobacteria*.

### EA regulates gut microbiota functions associated with lipid metabolism and endotoxin-mediated systemic inflammation

PICRUSt2-predicted functional KEGG pathway analysis revealed abnormal functional pathways in rotenone-treated rats, especially those related to lipid metabolism. Previous studies on microbiota function in human Parkinson’s disease patients have focused on loss of fatty acid metabolism and dysregulation of lipid metabolism, especially bile acid metabolism (Li et al., 2021), which is surprisingly consistent with our results (Figures 5B,C). Bile acids interact with their receptors to inhibit apoptosis, inflammation, and oxidative stress (Wahlstrom et al., 2016). However, regardless of its intermediate pathways, as a direct inducement of neuronal damage, the inflammation and oxidative stress have always been of concern in the central pathology of Parkinson’s disease. NOD-like receptors are associated with a variety of inflammatory diseases (Chen et al., 2009), and several of their family members are involved in the immune response process in *Shigella* and LPS (Girardin et al., 2001; Kim et al., 2008). The regulation of NOD-like receptor signaling pathways (Figure 5E), serum LPS (Figure 8C) and inflammatory factors (Figure 8D) suggests that EA may modulate systemic inflammation through gut microbiome and innate immune system.

Alterations in lipid species due to dysregulation of lipid-metabolizing enzymes may directly promote PD pathology (Xicoy et al., 2019). In particular, the hallmark pathological protein in PD, α-syn, has lipid membrane functions, and the endosome-lysosomal system and synaptic signaling pathways in PD are critically dependent on lipid dynamics (Galper et al., 2022). Moreover, PD genetics suggests that dysregulation of lipid homeostasis may contribute to the development of disease (Fanning et al., 2020; Fais et al., 2021). For example, β-glucocerebrosidase (GBA), which encodes glucocerebrosidase, a lysosomal hydrolase of lipid metabolism, is the most common genetic factor that increases the risk of PD (Aharon-Peretz et al., 2004). Thus, lipids are a promising research avenue to understand the etiology of PD as well as potential pharmacodynamic biomarkers of PD. Lipid metabolism is mainly regulated by nutrients like sugars and fatty acids. Studies have shown that therapies targeting the gut microbiota can improve metabolic function (Koutnikova et al., 2019). However, several reports have shown that lipid levels are relevant with the gut microbiota composition and microbial metabolites (Fu et al., 2015; Just et al., 2018). The influence of the gut microbiota on host lipid metabolism may be mediated through microbial metabolites generated by the gut microbiota, including SCFA, secondary bile acids, trimethylamine and LPS (Schoeler and Caesar, 2019). In addition, microbiota-induced changes in the lipid composition of the host cell membrane can affect signaling pathways, and the resulting downstream products can affect host local tissue and systemic immunity and metabolism (Brown et al., 2023). Gut dysbiosis is associated with metabolic disorders, and there exists a causal relationship between microbial function and metabolic disorders. However, the mechanistic links between gut microbiota and lipid metabolism and how the specific lipids affect microbial profile remains elusive.

Recent investigations have suggested that acupuncture also modulated various intestinal microbial metabolites and metabolic pathways (Yang et al., 2022). According to our study, the KEGG pathway enrichment analysis performed on metabolic pathway showed that differential lipid metabolism pathways were significantly enriched between PD and EA groups. To further explore the relationship between gut microbiota and metabolites differences, we identified a total of 474 metabolites of gut microbiota with significant differences were detected in EA-treated rats compared with PD rats (Figure 6E). Among these metabolites, lipid metabolism, such as biosynthesis of unsaturated fatty acids, fatty acid and bile acid was differentially expressed (Supplementary Table S5). Furthermore, we selected the first 50 differential metabolisms by MSEA enrichment analysis (Figure 6D). EA particularly affected dopamine 3-O-sulfate, L-Gulonolactone, and S-adenosyl-L-homocysteine (Supplementary Table S5), which were reported from previous studies involved in the response to treatment in PD patients, respectively (Bronaugh et al., 1975; Muller and Kuhn, 2006; Harrison et al., 2008). Additionally, we identified those lipids derived from microbiota, including β-Glycerophosphoric acid, 4-Hydroxybenzoic acid, and 4-Methoxycinnamic acid (Supplementary Table S5). Recent studies of brain-penetrating polyphenolic acids, namely, 3-hydroxybenzoic acid (3-HBA), 4-hydroxybenzoic acid (4-HBA), 3,4-dihydroxybenzoic acid (3,4-diHBA), and 3-(3-hydroxyphenyl) propionic acid (3-HPPA), show ability to effectively modulate the development to PD-type neuropathy and the progression of α-synucleinopathy (Ho et al., 2019; Ono et al., 2020).

To further explore the correlation between the significantly different intestinal microbiota and lipid metabolism, we conducted Spearman correlation analysis. Our results showed that Most of the lipids were negatively correlated with *Escherichia-Shigella* and positively correlated with *Lactobacillus*, *Dubosiella* and *Bifidobacterium* (Figure 6G). Based on these results, we speculated that a possible correlation between the altered lipids metabolism and gut microbiota compositions would bring new avenues for targeting PD pathological process, which may be an important pathway for acupuncture to regulate the correlation between the microorganisms and metabolomics.

### EA reversal of SNpc lipid peroxidation is associated with correction of endotoxin-mediated systemic inflammation

In addition to serving as essential structural components of the cellular membranes, plasmalogens play many crucial roles in cellular functions, including reservoirs for second messengers and working as endogenous antioxidants (Nagan and Zoeller, 2001; Braverman and Moser, 2012). It has been demonstrated that aberrant metabolism of plasmalogens is closely associated with insulin resistance (Tonks et al., 2016), atherosclerosis (Rasmiena et al., 2015), neurodegeneration (i.e., Alzheimer’s disease, Parkinson’s disease) (Han et al., 2005; Han, 2010), and aging (Lessig and Fuchs, 2009), etc. Decreased plasmalogen contents with increased choline and ethanolamine lysoglycerophospholipids (particularly those containing PUFAs) indicate the increased oxidative stress (Gross, 1984). Our results show that pPE content in the Model group was significantly increased, while LPE content showed an increasing trend without statistical difference. This indicates that under oxidative stress, the body generated more plasmalogen to resist oxidation, so there is no systemic lipid peroxidation. However, the antioxidant capacity of SNpc does not seem to be effective. Significantly lower pPE and higher LPE levels (Figure 7D) suggest obvious oxidative stress in SNpc, and elevated 4-hydroxyalkenal species (Figure 7E) confirm the presence of lipid peroxidation. After EA intervention, both pPE and LPE were increased, indicating that EA intervention increased plasmalogens synthesis to combat oxidative stress, and lipid peroxidation was also controlled.

Moreover, correlation analyses also verified strong correlations of *Escherichia-Shigella* and *Morganella* with motor symptoms and SNpc lipid peroxidation. Dysbiosis of the microbiota leads to increased intestinal permeability, allowing the LPS product to be transferred from the intestinal lumen into the host circulation (Rietschel et al., 1994; Tilg et al., 2016), which corroborates with our results (Figure 8C). Different routes and modes of LPS administration can cause a variety of Parkinson’s disease symptoms. An animal experiment showed that injection of LPS into the rat substantia nigra activated microglia, triggered an inflammatory response, increased the expression of pro-inflammatory cytokines, and altered the activity of oxidative stress markers, inducing the characterization of a PD model (Sharma and Nehru, 2015). Intraperitoneal injection of LPS increases α-syn expression and intestinal permeability in the large intestine (Kelly et al., 2014). Thus, LPS is widely used in models of neuroinflammation and oxidative stress in Parkinson’s disease (Deng and Bobrovskaya, 2022). It has been shown that combinations of bacteria and their products rather than specific bacteria appear to be responsible for the specific folding of α-syn (Stolzenberg et al., 2017; Manfredsson et al., 2018; Terada et al., 2018). Therefore, the study of bacterial clusters with similar functional characteristics, such as LPS leakage, is more relevant than simply focusing on specific bacterial species. Future studies should not be limited to characterizing pathogenic bacteria, such as *Escherichia–Shigella*, or beneficial bacteria, such as *Lactobacillus*.

Due to conditions, no colony transplantation or *in vitro* culture experiments were performed to clarify that modulation of the gut microbiota is a necessary way for EA to intervene in the motor symptoms of PD. For example, EA at ST25 may also inhibit LPS-induced inflammation exertion by activating NPY^+^ peripheral sympathetic neurons projecting to immune organs such as the spleen However, Prof. Qiu-Fu Ma ‘s study showed that the activation of the sympathetic anti-inflammatory pathway by EA at ST25 is time-sensitive. In concrete terms, if high-intensity EA stimulation at ST25 is given before the onset of LPS-induced systemic inflammation, it can exert a β2-noradrenergic receptor-mediated anti-inflammatory effect through the activation of peripheral NPY^+^ sympathetic neurons; whereas, applying the same abdominal acupoints and stimulation intensities after the induction of LPS-induced inflammation, it exhibits a markedly pro-inflammatory effect, which is mainly due to the fact that LPS can induce an increase in expression of inflammation-promoting α2-adrenergic receptor (Liu et al., 2020). This somehow suggests that there must be other powerful and effective anti-inflammatory pathways. Considering the site specificity of ST25 and its ability to regulate intestinal motility functions (Song et al., 2022), there exists a particular value in the study of gut microbiota. Relative to acute inflammatory diseases such as sepsis, gut microbiota modulation, a mild and long-lasting anti-inflammatory tool, is better suited for chronic inflammatory diseases as opposed to autonomic anti-inflammatory pathways.

## Conclusion

This study suggests that the improvement of motor function in PD model by EA may be mediated in part by restoring the gut microbiota. Its intermediate processes involve circulating endotoxins and inflammatory mediators, SNpc oxidative stress and lipid peroxidation. In addition, the specific effect of EA on gut microbiota at the genus level may explain its powerful anti-Parkinson effect on PD model. It is suggested that the gut-microbiome – brain axis may be a potential mechanism of EA treatment for the PD rats.

## Data availability statement

The datasets presented in this study can be found in online repositories. The names of the repository/repositories and accession number(s) can be found at: https://www.ncbi.nlm.nih.gov/, PRJNA958618.

## Ethics statement

The animal study was approved by Institutional Animal Care and Use Committee of the Nanjing University of Traditional Chinese Medicine, Nanjing, China (permission no. 202112A047). The study was conducted in accordance with the local legislation and institutional requirements.

## Author contributions

X-mH: Conceptualization, Funding acquisition, Investigation, Methodology, Project administration, Writing – original draft. L-z-xS: Conceptualization, Investigation, Methodology, Project administration, Writing – original draft. Z-zZ: Data curation, Software, Visualization, Writing – original draft. XR: Data curation, Writing – review & editing. H-cL: Methodology, Writing – review & editing. ZY: Conceptualization, Investigation, Methodology, Writing – review & editing. LH: Conceptualization, Investigation, Methodology, Writing – review & editing.

## Footnotes

^1^ http://www.kegg.jp/kegg/compound/

^2^ http://www.kegg.jp/kegg/pathway.html

## Supplementary material

SUPPLEMENTARY TABLE S1 Goods coverage of all samples.

SUPPLEMENTARY TABLE S2 List of bacterial genera with significant differences in relative abundance between gut microbiota of Control, Model and EA groups.

SUPPLEMENTARY TABLE S3 Spearman correlation analysis of 68 genera and motor functions.

SUPPLEMENTARY TABLE S4 List of PICRUSt2-predicted functional KEGG pathways.

SUPPLEMENTARY TABLE S5 List of differential metabolites Information.

SUPPLEMENTARY TABLE S6 Spearman correlation analysis of 68 genera and TH+ neurons or 4-HNE.

SUPPLEMENTARY DATA SHEET 1 Figure S1: Evaluation of modelling.

SUPPLEMENTARY DATA SHEET 2 Figure S2: 16S rRNAseq Rarefaction curve, Shannon-Wiener curve, species accumulation curves.

SUPPLEMENTARY DATA SHEET 3 Figure S3: Diversity analysis of intestinal flora.
