# Supplementary material for: Electroacupuncture at ST25 corrected gut microbial dysbiosis and SNpc lipid peroxidation in Parkinson’s disease rats
Source: Front Microbiol. 2024 Feb 21;15:1358525. doi: 10.3389/fmicb.2024.1358525 (PMC10915097; doi:10.3389/fmicb.2024.1358525)
Supplement: SUPPLEMENTARY TABLE S2 — List of bacterial genera with significant differences in relative abundance between gut microbiota of Control, Model and EA groups. [file Table_2.DOCX]

**Table S2.** List of bacterial genera with significant differences in relative abundance between gut microbiota of Control, Model and EA groups.

| **Genus** | **Control** | **Model** | | **EA** | | ***FDR P*** |
| --- | --- | --- | --- | --- | --- | --- |
| *Saccharofermentans sp* | 0±0 | 0±0 |  | 0.001007±0.000585 |  | 0.019 |
| *Dorea* | 0.000883±0.000277 | 0.001368±0.00032 |  | 0.000039±0.000019 | ### | 0.030 |
| *Ruminococcus gauvreauii group* | 0.001014±0.000283 | 0.001538±0.000194 |  | 0.000142±0.000031 | #### | 0.030 |
| *Coriobacteriaceae UCG-002* | 0.001233±0.000207 | 0.005118±0.002588 |  | 0.021451±0.006581 | # | 0.034 |
| *Subdoligranulum* | 0.010375±0.004091 | 0.016967±0.006874 |  | 0.000443±0.00019 | # | 0.034 |
| *Fournierella* | 0±0 | 0±0 |  | 0.000188±0.00006 | ## | 0.019 |
| *Eubacterium ruminantium group* | 0.000188±0.000066 | 0.000319±0.000101 |  | 0.000007±0.000007 | ## | 0.035 |
| *Faecalibaculum* | 0.000868±0.000487 | 0.001421±0.000879 |  | 0.005813±0.001675 | # | 0.045 |
| *Desulfoglaeba* | 0.000025±0.000014 | 0.000131±0.000065 |  | 0±0 | # | 0.049 |
| ***Morganella*** | 0.000014±0.000004 | 0.00005±0.000014 | * | 0±0 | ## | 0.030 |
| *Porphyromonas* | 0.000014±0.000007 | 0.000021±0.000011 |  | 0.009407±0.005869 |  | 0.030 |
| *Fusicatenibacter* | 0.004533±0.001391 | 0.005547±0.001923 |  | 0.00017±0.000087 | ## | 0.045 |
| ***NK4A214 group*** | 0.002743±0.000395 | 0.00481±0.000959 | * | 0.001152±0.000117 | ## | 0.030 |
| *Roseburia* | 0.018296±0.00446 | 0.015007±0.002172 |  | 0.001233±0.000399 | #### | 0.030 |
| *Rikenella* | 0±0 | 0±0 |  | 0.000156±0.000048 | ## | 0.019 |
| *Blautia* | 0.037404±0.008026 | 0.060305±0.010677 |  | 0.001443±0.000347 | ### | 0.030 |
| *Bilophila* | 0.0014±0.000644 | 0.000836±0.000138 |  | 0.000025±0.000017 | ### | 0.030 |
| *Campylobacter* | 0±0 | 0±0 |  | 0.000156±0.000119 |  | 0.030 |
| ***UCG-003*** | 0.000454±0.00013 | 0.000847±0.000094 | * | 0.000124±0.000044 | #### | 0.030 |
| ***Escherichia-Shigella*** | 0.006426±0.001472 | 0.027115±0.006894 | ** | 0.002984±0.000887 | ## | 0.030 |
| ***Lachnospiraceae UCG-008*** | 0.001116±0.000403 | 0.003477±0.000745 | ** | 0.000174±0.000091 | ### | 0.030 |
| *CHKCI001* | 0±0 | 0±0 |  | 0.000152±0.000087 |  | 0.030 |
| *Pygmaiobacter* | 0.000043±0.000016 | 0.00017±0.000075 |  | 0±0 | # | 0.030 |
| *Colidextribacter* | 0.00587±0.000872 | 0.012278±0.003393 |  | 0.002768±0.000577 | ## | 0.032 |
| *Erysipelatoclostridium* | 0.001198±0.000217 | 0.001499±0.000309 |  | 0.000415±0.000124 | ## | 0.042 |
| ***Adlercreutzia*** | 0.000337±0.00007 | 0.001109±0.000246 | ** | 0.000206±0.00005 | ## | 0.030 |
| *Erysipelothrix* | 0±0 | 0±0 |  | 0.001879±0.001318 |  | 0.042 |
| *Lachnospira* | 0.000074±0.000031 | 0.000447±0.000283 |  | 0±0 |  | 0.030 |
| *Pyramidobacter* | 0±0 | 0±0 |  | 0.006143±0.003969 |  | 0.019 |
| *Corynebacterium* | 0.000149±0.000086 | 0.000252±0.000195 |  | 0.002059±0.000578 | ## | 0.035 |
| *Parabacteroides* | 0.012452±0.002428 | 0.011888±0.004953 |  | 0.001677±0.000521 | # | 0.035 |
| ***Bacteroides pectinophilus group*** | 0.000131±0.000042 | 0.000326±0.000068 | * | 0±0 | ### | 0.030 |
| *Collinsella* | 0.001964±0.000768 | 0.002329±0.000633 |  | 0.000113±0.000073 | ## | 0.032 |
| *Agathobacter* | 0.000308±0.000213 | 0.000443±0.00013 |  | 0.000014±0.000009 | ## | 0.035 |
| ***Dubosiella*** | 0.00095±0.00026 | 0.005827±0.002116 | * | 0.118547±0.042696 | ## | 0.026 |
| *Gemella* | 0.000057±0.000012 | 0.000053±0.000024 |  | 0±0 | # | 0.032 |
| *Lactobacillus* | 0.096065±0.031898 | 0.04534±0.008408 |  | 0.224689±0.041857 | ### | 0.032 |
| *Eisenbergiella* | 0.000025±0.00001 | 0.000025±0.000013 |  | 0.000213±0.000068 | ## | 0.035 |
| ***sulfate-reducing bacterium KNH*** | 0±0 | 0.000021±0.00001 | * | 0±0 | # | 0.042 |
| *Eubacterium siraeum group* | 0.000071±0.000038 | 0.000248±0.000129 |  | 0.000805±0.00039 |  | 0.034 |
| *Pseudomonas* | 0.001042±0.000703 | 0.000783±0.000148 |  | 0.04152±0.010984 | ## | 0.030 |
| ***Candidatus Soleaferrea*** | 0.000014±0.000011 | 0.000106±0.000011 | **** | 0.000028±0.000013 | ### | 0.030 |
| *Citrobacter* | 0.000046±0.000034 | 0.000698±0.000393 |  | 0.000004±0.000004 |  | 0.042 |
| *Lachnospiraceae UCG-001* | 0.00017±0.000025 | 0.000113±0.00002 |  | 0.00005±0.000014 | # | 0.044 |
| *mouse gut metagenome* | 0.000057±0.000011 | 0.000085±0.00003 |  | 0.003084±0.000767 | ### | 0.030 |
| *Eggerthella* | 0.000167±0.000068 | 0.000344±0.000117 |  | 0±0 | ## | 0.032 |
| ***Monoglobus*** | 0.003318±0.000615 | 0.005834±0.000999 | * | 0.001138±0.000142 | ### | 0.030 |
| *Allobaculum* | 0.002081±0.000385 | 0.00587±0.003028 |  | 0.009006±0.001966 |  | 0.049 |
| *Anaerovorax* | 0.000408±0.000105 | 0.000638±0.000109 |  | 0.000167±0.000027 | ### | 0.039 |
| *Rodentibacter* | 0.001613±0.001017 | 0.00033±0.000063 |  | 0.000082±0.000022 | ## | 0.035 |
| *UBA1819* | 0.001085±0.000191 | 0.000904±0.000119 |  | 0.000057±0.000017 | #### | 0.030 |
| *Candidatus Stoquefichus* | 0.002102±0.001461 | 0.001407±0.000872 |  | 0±0 |  | 0.030 |
| *Helicobacter* | 0.000004±0.000004 | 0.000004±0.000004 |  | 0.002003±0.001181 |  | 0.030 |
| *Peptostreptococcus* | 0±0 | 0±0 |  | 0.000035±0.000014 | # | 0.042 |
| *Gallibacterium* | 0±0 | 0±0 |  | 0.007302±0.002851 | # | 0.019 |
| ***GCA-900066575*** | 0.000968±0.000175 | 0.002084±0.000458 | * | 0.000351±0.00011 | ## | 0.030 |
| *Parasutterella* | 0.006483±0.001472 | 0.00689±0.001779 |  | 0.001701±0.00062 | ## | 0.039 |
| *Erysipelotrichaceae UCG-003* | 0.001457±0.000631 | 0.003172±0.000973 |  | 0±0 | ## | 0.030 |
| *Alloprevotella* | 0.001425±0.000405 | 0.001783±0.000668 |  | 0.000188±0.000053 | # | 0.042 |
| ***Family XIII AD3011 group*** | 0.000712±0.000165 | 0.001623±0.000192 | ** | 0.000588±0.00014 | ### | 0.035 |
| *Eubacterium hallii group* | 0.011225±0.004262 | 0.009942±0.002354 |  | 0.000035±0.000014 | ### | 0.030 |
| ***UCG-008*** | 0.001049±0.000231 | 0.000532±0.000068 | * | 0.000032±0.00002 | #### | 0.030 |
| *Oscillibacter* | 0.001389±0.000316 | 0.001545±0.000443 |  | 0.00034±0.000086 | ## | 0.050 |
| *UCG-002* | 0.000507±0.000419 | 0.001159±0.000438 |  | 0.000004±0.000004 | ## | 0.035 |
| *Klebsiella* | 0.002892±0.001547 | 0.004221±0.002123 |  | 0.000032±0.000017 | # | 0.035 |
| ***Tuzzerella*** | 0.000517±0.00012 | 0.00106±0.000148 | ** | 0.000237±0.000039 | ### | 0.030 |
| *Ruminococcus gnavus group* | 0.001889±0.001317 | 0.001163±0.0006 |  | 0.000014±0.000011 | # | 0.032 |
| *Akkermansia* | 0.016829±0.008719 | 0.003754±0.001927 |  | 0.000759±0.000499 |  | 0.042 |

All values are presented as means ± SEM. The genera are listed in order of significance (FDR *P* <0.05, Kruskal-Wallis test). Genera in bold are those showing significant difference between Model and Control groups (*p* <0.05, Metastats).
