# Supplementary material for: Electroacupuncture at ST25 corrected gut microbial dysbiosis and SNpc lipid peroxidation in Parkinson’s disease rats
Source: Front Microbiol. 2024 Feb 21;15:1358525. doi: 10.3389/fmicb.2024.1358525 (PMC10915097; doi:10.3389/fmicb.2024.1358525)
Supplement: SUPPLEMENTARY DATA SHEET 1 — Figure S1: Evaluation of modelling. [file Data_Sheet_1.PDF]

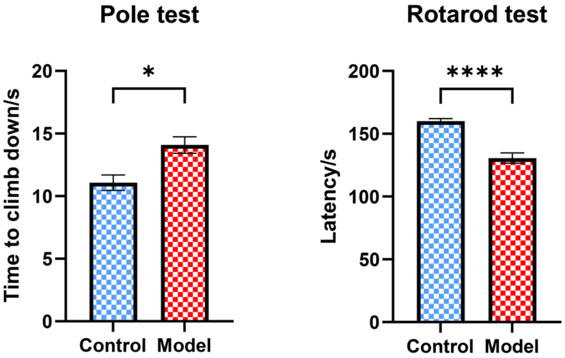


Figure S1 Evaluation of modeling
Pole and rotarod tests at the end of the fourth week of modeling. (n=6/12, Student t-test, * $P < 0.05$, **** $P < 0.0001$).
