# Supplementary material for: Electroacupuncture at ST25 corrected gut microbial dysbiosis and SNpc lipid peroxidation in Parkinson’s disease rats
Source: Front Microbiol. 2024 Feb 21;15:1358525. doi: 10.3389/fmicb.2024.1358525 (PMC10915097; doi:10.3389/fmicb.2024.1358525)
Supplement: SUPPLEMENTARY DATA SHEET 2 — Figure S2: 16S rRNAseq Rarefaction curve, Shannon-Wiener curve, species accumulation curves. [file Data_Sheet_2.PDF]

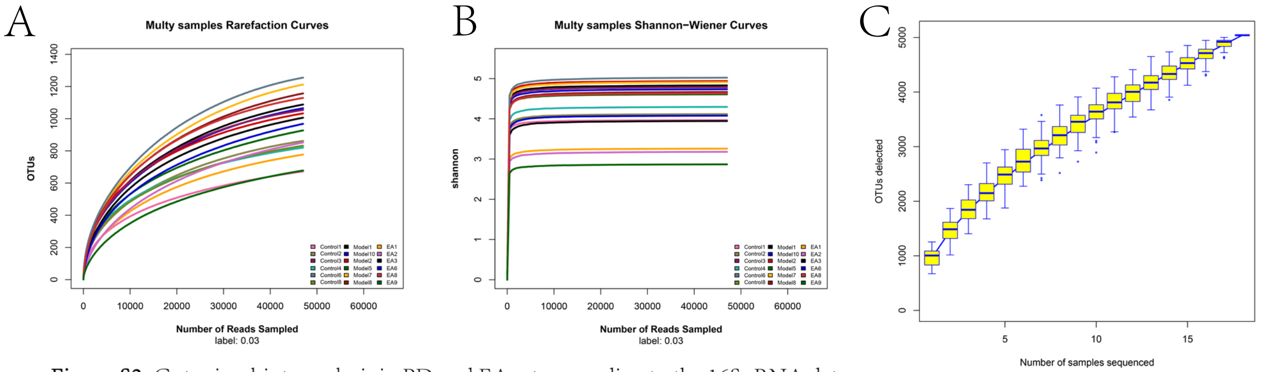

Figure S2. Gut microbiota analysis in PD and EA rats according to the 16S rRNA data.

A. Rarefaction curves generated from the OTUs .

B. Shannon-Wiener curves generated from the OTUs . Abscissa is sequencing depth and ordinate is shannon index.

C. Species accumulation curves. The abscissa is the sample size, and the ordinate is the number of OTUs after sampling. The abscissa is the amount of randomly extracted sequencing data, and the ordinate is the number of OTUs observed.

The above three figures suggested that high sampling coverage (⩾99%) was achieved in all sample.
