# Supplementary material for: Electroacupuncture at ST25 corrected gut microbial dysbiosis and SNpc lipid peroxidation in Parkinson’s disease rats
Source: Front Microbiol. 2024 Feb 21;15:1358525. doi: 10.3389/fmicb.2024.1358525 (PMC10915097; doi:10.3389/fmicb.2024.1358525)
Supplement: SUPPLEMENTARY DATA SHEET 3 — Figure S3: Diversity analysis of intestinal flora. [file Data_Sheet_3.PDF]

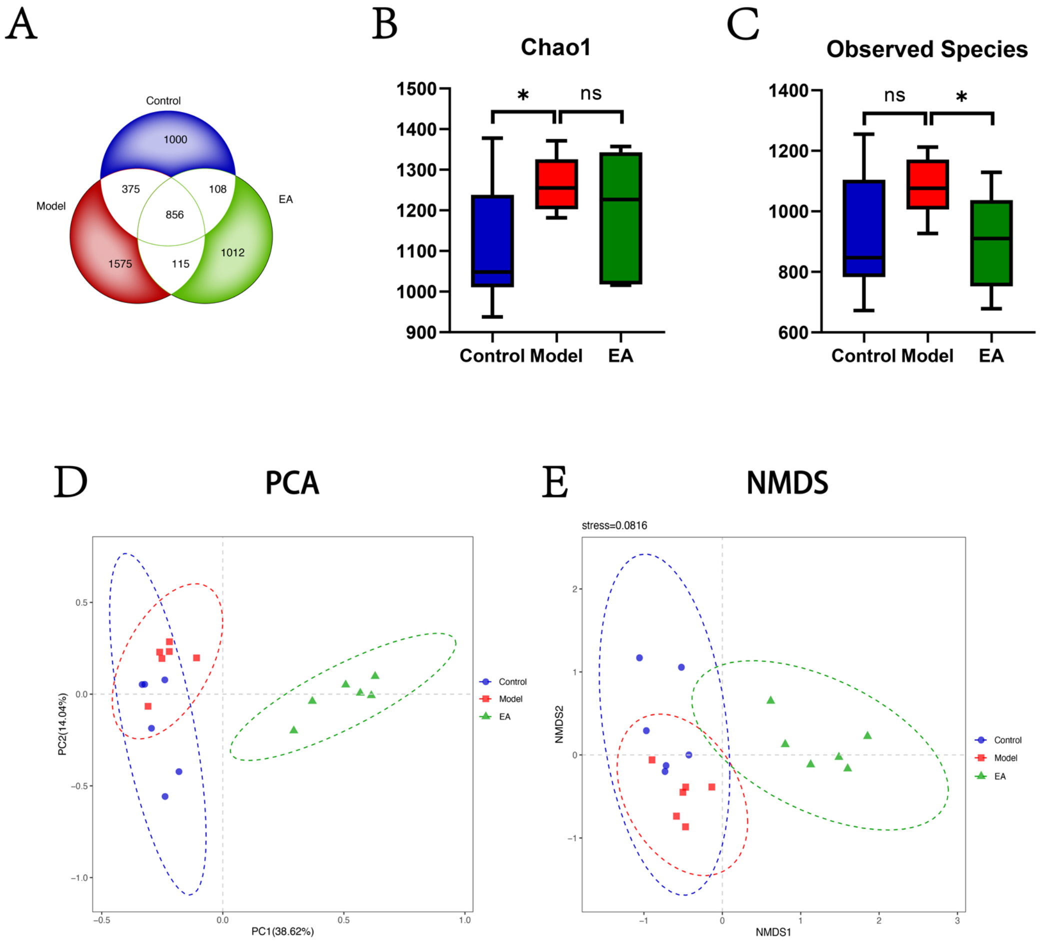

Figure S3 Diversity analysis of intestinal flora. **(A)**Venn diagram of the observed OTUs. **(B)**Rotenone treatment increased Chao1 of the Model group.**(C)** Compared with the Model group, EA decreased Observed Species(n = 6, Student t-test, *P < 0. 05). **(D)**PCA and **(E)**NMDS (stress=0.0816) analysis showed that the bacterial communities in each group were far apart and clearly demarcated.
